# Hypoglicemic and Hypolipedimic Effects of *Ganoderma lucidum* in Streptozotocin-Induced Diabetic Rats

**DOI:** 10.3390/medicines5030078

**Published:** 2018-07-28

**Authors:** Erna Elisabeth Bach, Edgar Matias Bach Hi, Ana Maria Cristina Martins, Paloma A. M. Nascimento, Nilsa Sumie Yamashita Wadt

**Affiliations:** 1 Biomedical Sciences, Health Department, UNINOVE (Universidade Nove de Julho), São Paulo 01156-050, São Paulo, Brazil; 2 Experimental Biochemistry Academic Nucleum (NABEX), UNILUS (Lusiada University Center), Santos 11050-071, São Paulo, Brazil; edgarbach@gmail.com; 3 Biological Institute, São Paulo 04014-900, São Paulo, Brazil; crisfm@biologico.sp.gov.br; 4 Biomedical Sciences, Health Department, Scientific Research Student, UNINOVE (Universidade Nove de Julho), São Paulo 01156-050, São Paulo, Brazil; andrade.nascimento@bol.com.br; 5 Pharmacy, Health Department, Sciences, UNIP (Universidade Paulista), Jundiaí 13214-525, São Paulo, Brazil; nilwadt@gmail.com

**Keywords:** diabetes, ganoderma, beta-glucan, phenols, kynurenine, tryptophan

## Abstract

**Background:***Ganoderma lucidum* (Leyss. Ex. Fr) Karst is a basidiomycete mushroom that has been used for many years as a food supplement and medicine. In Brazil, National Health Surveillance Agency (ANVISA) classified *Ganoderma lucidum* as a nutraceutical product. The objective of the present work was to observe the effects of an extract from *Ganoderma lucidum* in rats treated with streptozotocin, and an agent that induces diabetes. **Method**: Male Wistar rats were obtained from the animal lodging facilities of both University Nove de Julho (UNINOVE) and Lusiada Universitary Center (UNILUS) with approval from the Ethics Committee for Animal Research. Animals were separated into groups: (1) C: Normoglycemic control water; (2) CE: Normoglycemic control group that received hydroethanolic extract (GWA); (3) DM1 + GWA: Diabetic group that received extract GWA; and (4) DM1: Diabetic group that received water. The treatment was evaluated over a 30-day period. Food and water were weighted, and blood plasma biochemical analysis performed. **Results**: *G. lucidum* extract contained beta-glucan, proteins and phenols. Biochemical analysis indicated a decrease of plasma glycemic and lipid levels in DM rats induced with streptozotocin and treated with GWA extract. Histopathological analysis from pancreas of GWA-treated DM animals showed preservation of up to 50% of pancreatic islet total area when compared to the DM control group. In plasma, Kyn was present in diabetic rats, while in treated diabetic rats more Trp was detected. **Conclusion**: Evaluation from *G. lucidum* extract in STZ-hyperglycemic rats indicated that the extract possesses hypoglycemic and hypolipidemic activities. *Support: Proj. CNPq 474681/201*.

## 1. Introduction

*Ganoderma lucidum* (Fr.) Krast, a basidiomycete belonging to the *Ganodermataceae* family, is one of the most famous traditional Chinese medicinal herbs, which has been used as a food and in folk medicine [1]. Fruiting bodies of these fungi are naturally produced but are not sufficient for commercial use [2], however, using the Jun-Cao farming method, it is possible to achieve a volume able to supply the market demand [3]. In Brazil, some people have produced this fungus using the Jun-Cao method, and in the country, it is classified as a nutraceutical product by ANVISA (National Agency of Health Surveillance) [4]. The mushroom is a popular folk medicine for the prevention or treatment of diseases such as hepatitis, hypertension, hypercholesterolemia, arthritis and bronchitis. In addition, *G. lucidum* presents a variety of biological activities, such as: immunostimulant, antimicrobial, anti-inflammatory, anti-hyperglycemic, hepatic and renal protection, antioxidant and antiviral effects [5,6,7]. Different kinds of bioactive polysaccharides have been extracted and isolated from the fruiting bodies of different *Ganoderma* species and represent a structurally diverse class of biological macromolecules with a wide-range of physiological properties. Wadt et al. [8] described the presence of beta glucan, proteins, water soluble heteropolysaccharides and phenols in extracts of *Ganoderma lucidum* from São Paulo.

The objective of the present work was to evaluate the effect of chemical compounds found in *Ganoderma lucidum* for rats with streptozocin-induced diabetes mellitus.

## 2. Material and Methods

### 2.1. Extract from *Ganoderma lucidum*

The fruiting bodies of *G. lucidum* were collected in São Paulo, Brazil and after dried, were finely milled in a hammer mill equipped with a 1 mm mesh stainless steel sieve.

Powdered mushroom was submitted to two methods for extracting: Extract 1-mass homogenate (100 g) in 100 mL of water, kept at 60 °C for one hour (GHotW) and then filtered with paper filter; Extract 2–50 g of *G. lucidum* was percolated with alcohol 70% (GAlc) for one week, filtered, fully extracted and concentrated until the volume of 50 mL, and kept in bottles until further use.

For oral administration, the GWA extract (GWater–Alcohol or Hydroethanolic extract) was obtained from a mixture of 80% of Extract 1 and 20% of Extract 2. In a work previously published by this group, Wadt et al. [8] described that hydroethanolic extract (GWA) did not exhibit acute toxicology in mice.

Extract was submitted to proteins [9], phenols [10] and beta-glucan quantification by Lever method [11]. After quantifying, phenols and ganoderic acid were analyzed by HPLC. Phenolic compounds and Ganoderic acid were separated in a High performance liquid chromatograph (HPLC) system (model Young Lin YL 9300, Young Lin Bldg, Hogye-dong, Anyang, Korea) equipped with a quaternary gradient pump unit, an UV–vis detector and the column oven (YL9330, Young Lin Bldg, Hogye-dong, Anyang, Korea). The analytical column used was a Kinetex C18 (Phenomenex) (4.6 mm × 250 mm i.d., 5 um). The wavelength for UV detection was 254 nm. Elution was carried out at a flow rate of 1.0 mL/min at 35 °C. The mobile phase A consisted of methanol and the mobile phase B was 0.1% of acetic acid in water. The injection volume was 20 uL. The standart phenolic compounds were purchased from Sigma (Merck, Rio de Janeiro, Brazil) (coumaric acid, ferulic acid, rutin, cafeic acid, quercetin, kaempferol) and dissolved in HPLC grade (methanol). Ganoderic acid A (AcG) from Sigma was dissolved in methanol, achieving final concentration of 100, 50 and 25 ug/mL. For identification was used specific retention times and the peak areas were automatically measured by software Clarity, obtaining the calibration curve.

### 2.2. Animals Testing

Male Wistar rats aged four weeks and weighing 250 to 300 g were obtained from bioterium of both University Nove de Junho (UNINOVE) and Lusiada Universitary Center (UNILUS). The animals were kept in polypropylene cages (two to three animals per cage) covered with metallic grids in a room maintained at 23 °C, 55 ± 10% relative humidity and a 12 h light/dark cycle. The animals had free access to food and water for two weeks before beginning the study. The UNINOVE and UNILUS Ethics Committee for Animal Research approved the protocols used in this study (UNINOVE process numbers: 34/2010 and 20/2012; UNILUS process number: 02/2012). All animals were weighed during the experiment.

### 2.3. Diabets with Streptozotocin

Animals were divided into four groups of five rats. In two groups, the animals were fasted for 12 hours and chemical diabetes was induced through an intraperitoneal injection of streptozotocin (50 mg/kg) (STZ, Sigma). The STZ solution was prepared immediately prior injection by dissolving the drug in a fresh, cold citrate buffer, pH 4.5. After 72 h, blood glucose levels were measured using a portable glucose meter (One Touch II; Johnson and Johnson, São Paulo, Brazil). For such, the distal part of the tail was gently snipped; the first blood drop was discarded and the second was absorbed by a test strip inserted in the glucose meter. Rats were considered diabetic when the blood glucose level was at least 250 mg/dL. To avoid possible hypoglycemia, the animals received oral solution of dextrose 5% *ad libtum* only for the first 48 h after induction.

Groups of rats were separated as following: (1) C: Normoglycemic control that received water by gavage; (2) CE: Normoglycemic control group that received GWA extract; (3) DM1 + GWA: Diabetic group that received extract; and (4) DM1: Diabetic group that received water, with dose of (1 mL/Kg/day) for all groups. The groups were evaluated for 30 days. Food and water consumption were measured every two days. Blood glucose levels and weight were evaluated twice a week, always at 11:00 a.m. At the end of the experimental period, the animals were euthanized with a lethal dose of a cocktail containing ketamine (1 mg) and xylazine (5 mg). Thoracotomy was performed. Blood was collected from the left ventricle and centrifuged. The plasma was removed and stored at −20 °C for no longer than three days before the assay. Total cholesterol, triglycerides, urea and creatinine were measured using test kits (Labtest Diagnostica, Lagoa Santa, Minas Gerais, Brazil). The variance analysis was performed by the One Way ANOVA test, following T Student Test (*p* < 0.05).

### 2.4. Histopathology

After euthanasia, pancreas was removed, rinsed with water, fixed in a 10% buffered formalin solution and embedded in paraffin wax. Sections measuring 5 μm in thickness were prepared and stained with hematoxylin and eosin. The three best sections from each animal were submitted to microscopic examination. Five non-consecutive areas of each sample were photographed in microscope with digital camera and polarizer (Carl Zeiss, model Axion Scope A1, Oberkochen, Germany) and analyzed using the software (Carl Zeiss, Zen 2012, Oberkochen, Germany).

### 2.5. Evaluation of Amino Acid in Plasma

500 uL of plasma from each animal was precipitated with ammonium sulfate up to 60%, to remove the globulin, remaining over-night in the refrigerator. After centrifugation (3400 rpm, 5 min, eppendorf), precipitates were resuspended in phosphate buffer pH = 7 and dialyzed (Tubbing MW 10,000 daltons) for the removal of the salt used. Samples were filtered in millipore 0.25 um, and 20 uL was injected into HPLC. YL-9300 UV-coupled HPLC instrument 254 nm, C18 Luna 25 cm × 4.5 mm reverse phase column, temperature 27–30 °C. Separation was done in the following mobile phase: buffer sodium acetate 10 mM in MilliQ water (A) and acetonitrile (B): 0–1 min (20% B); 1.01–1.5 min (5% B); 1.51–8 min (4% B). The flow rate was kept constant at 1 mL/min and peaks were detected at 254 nm. All chemicals reagents used in the analysis, such as acetonitrile and acetate buffer we purchased from Sigma and Merck, (Rio de Janeiro Brazil), with high purity levels. The standards used were Kynerunin-Kyn (20 uM), Tryptophan-Trp (20 uM), Cysteine-Cys (10 mM), Arginine-Arg (10 mM), Metionine-Met (10 mM), Proline-Pro (10 mM), Valine-Val (10 mM), Alanine-Ala (10 mM), Phenilalanine-Phe (10 mM), Tyrosine-Tyr (10 mM) from Sigma and Merck. All were evaluated and separated by HPLC based on the peak at said retention time (Rt) and height peak in millivolt (mV) or concentration in uM or ug.

## 3. Results

### 3.1. Biochemical Analysis

Basidiocarp form has colored pileus (brown) and concentric zones on the surface of the pileus that can identify *G. lucidum*. The fruiting body is rigid and extraction with hot water is important, for elicitation of polysaccharide along with phenols. Wadt et al. [8] demonstrated that the hydroethanolic extract has a high concentration of beta-glucan along protein and phenol (Table 1), added the fact that the dose assessed was not toxic to animal.

By HPLC analysis, we verified the presence of coumaric acid (1.46 ug/mL); 0.20 ug/mL of cafeic acid; 18.19 ug/mL of ferulic acid; 2.68 ug/mL of Kercetin; 12.21 ug/mL of rutin and 15.79 ug/mL of ganoderic acid in the extract.

### 3.2. Animals with Diabetes

**Figure 1:**
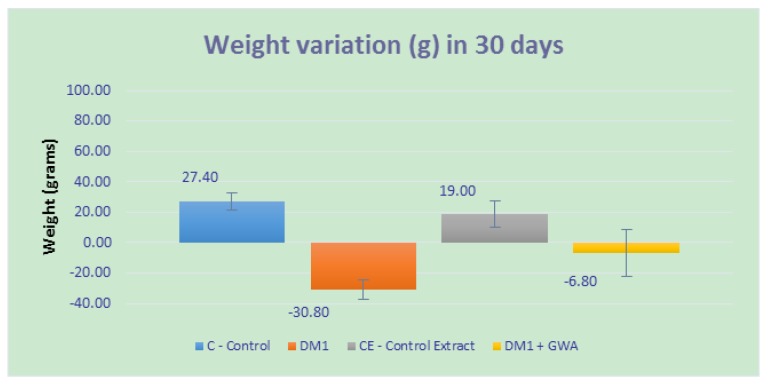
Weight variation (in grams) from animals since beginning of treatment until experimental end. *p* < 0.05 between C and CE groups; *p* < 0.01 between DM1 and DM1 + GWA (hydroethanolic extract) groups.

During the experiment (30 days), the animals were submitted to weighing at different times, and Figure 1 displays the variation between weights. The animals in the control group (C) had a weight gain around 27.4 g, while the group that received only GWA extract (in figure CE) had an increase of only 19 g. Animals from DM1 + GWA groups had a weight loss of 6.8 g when compared with DM1 animals, where the major loss is around 30.80 g.

**Figure 2:**
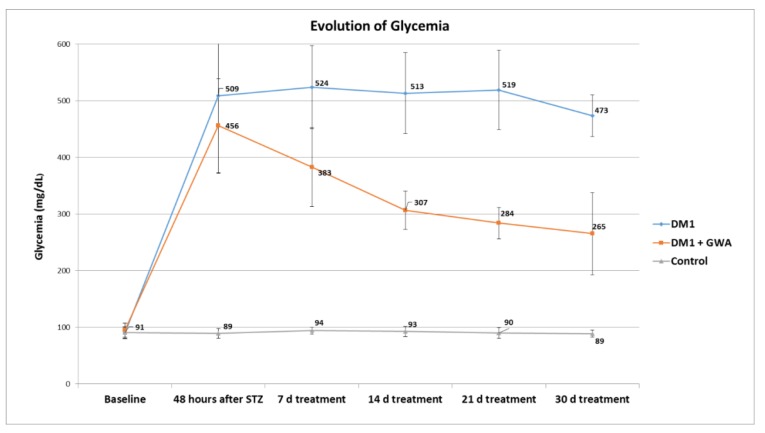
Blood glucose levels evolution during 30 days from Control, DM1 and DM1 + GWA groups. *p* < 0.01 between DM1 and DM1 + GWA groups.

When glycemia is observed, *Ganoderma* extract presented a blood glucose level decreasing effect within the first 48 h after treatment and diminishing over time until the end of 30 days (Figure 2).

Biochemical analysis also showed an effect of a decrease of total cholesterol, triglycerides (tendency), urea and creatinine in rats treated with *Ganoderma* and inoculated with Streptozotocin (Table 2). Histopathological analysis from pancreas of GWA-treated DM animals showed preservation of up to 50% of pancreatic islet area when compared to DM control group (Table 3).

### 3.3. Evaluation of Amino Acid in Plasma

Amino acid patterns were separated on HPLC (YL-9300) where they showed different retention times (Rt), following the mobile phase and method temperature, such as: Trp = 2.18 min; Kyn (Kynurenin) = 2.09 min; Cys = 2.73 min; Arg = 2.83 min; Met = 3.00 min; Pro = 4.25 min; Val = 4.38 min; Ala = 4.41 min; Phe = 4.55 min; Tyr = 4.65 min. Observing the plasma content of the animals after precipitation, dialysis and filtration along with the HPLC, several peaks were obtained, however, only 2 Rt were recognized among the standards of amino acids among others unknown.

The amino acid Trp was detected in all groups of this study. However, its concentration was higher in groups treated with GWA extract. In the CDM group, Trp concentration decreased, revealing the presence of Kyn (Table 4).

## 4. Discussion

Beta glucan is a bioactive substance found in *G. lucidum*, which is considered a nutraceutical food by ANVISA [4]. ANVISA by resolution (Resolution RDC nº 2, of 7 of January of 2002) had included beta-glucans with the following allegation: “The beta glucan (alimentary fiber) assists in the reduction of the absorption of the cholesterol. Its consumption must be associated to a balanced diet and healthful habits of life”. It is possible to commercialize the fungi in dust, capsule, tablet, being sold as a nutritional supplement in Brazil.

In hydroalcoholic extract of *Ganoderma*, were found beta glucan, protein and phenols. Many of these phenols act as antioxidant, protecting cells from many metabolic disturbs. Within these, we found in the extract: coumaric acid (1.46 ug/mL); 0.20 ug/mL of cafeic acid; 18.19 ug/mL of ferulic acid; 2.68 ug/mL of kercetin; 12.21 ug/mL of rutin and 15.79 ug/mL of ganoderic acid. Therefore, the extract was subjected in rats to evaluate a possible diabetes control. Wadt et al. [8] showed that the hydroethanolic extract of *Ganoderma* did not presented toxicity in a dosage of 1 mL/Kg for mice in an acute toxicology assay. There was no significant reduction in body mass, water and feed consumption between the treated and control groups, indicating the safe use of the hydroethanolic extract of *Ganoderma lucidum*.

Diabetes mellitus (DM) is a plurimetabolic disease of multiple etiology. The long-term effects of this disease include lesions, dysfunction or even failure of multiple organs. The disease is also a public health issue. Metabolic controls of individuals with the evolving disorder contribute to one of the biggest challenges for Brazil’s public health services, and worldwide [12]. Many authors describe the use of fungi with medicinal properties for the control of the disease and its complications [13,14].

Regarding the animal model used, many studies have proven that the induction of DM by STZ is an effective method to investigate the mechanism of disease [15]. Considering that *Diabetes mellitus* is a genetic disease, and that the physiopathological alterations delay appearance, the usage of experimental models for its fast induction has been increasingly considered [16]. According to Delfino et al. [17], a dose of 60 mg/kg of intraperitoneal STZ has shown to be very effective, and the objective of DM1 induction was achieved. This success was proven when blood glucose levels from induced groups were, after 48 h, 509 mg/dL (DM1 Control group) and 456 mg/dL (DM1 *Ganoderma* group), therefore showing a significant increase (*p* < 0.01) when compared to the non-induced group.

The treatment with 1.0 mL/day of *Ganoderma lucidum* showed to be effective, resulting in a significant decrease in blood glucose levels in the diabetic groups. After 1 week of treatment, the treated group had its glucose levels reduced, from 456 mg/dL to 383 mg/dL, which corroborates with results found by Seto et al. [18] that after the same period, with administration of 0.3 g/kg of *Ganoderma lucidum* extract, the glycemic value decreased from 528 mg/dL to 428 mg/dL.

This response to treatment may be related to the beta-glucans in *G. lucidum*, which have the capability to form an aqueous layer in the intestine, reducing the absorption of both sugars and lipids [19].

However, *G. lucidum*’s actions are not limited to glycemic levels. In relation to kidney profile, creatinine, after treatment, showed a significant decrease when compared to the diabetic control group. The same happened with urea. This might be explained by the evidences that *G. lucidum* delays the harmful effects of DM in kidneys and protects the renal tissue from the possible toxic effects of STZ. Cholesterol levels also had good results, displaying a significant decrease when compared with the DM Control group.

The morphological evaluation of the pancreatic islets revealed compensatory hyperplasia induced by *Ganoderma*. This finding suggests a stimulatory effect on these cells. These results agree with those observed by Mascaro et al. [20], when using *Agaricus* as a diabetes control.

Blood plasma is a rich source of biochemical products that can indicate physiological or clinical status of a patient or animal [21,22]. JIANG et al. [23] have fractionation of plasma proteins with 50% and 70% ammonium sulphate to reduce interfering substances and improve extraction, thereby facilitating the identification of disease markers. Tryptophan (Trp) is considered an essential amino acid for humans, it is the precursor of proteins and important in regulating molecules, such as serotonin [24] among others. However, it is believed that about 90% of the peripheral metabolic pathways involving Trp terminate in the production of the so-called kynurenin (Kyn) [25], where ammonium sulfate was used for precipitation at 60% and dialysis. Results of HPLC demonstrated that diabetic animals present Kyn in higher concentrations when compared with the control group, and when treated with Ganoderma, Trp level was increased in this group. That indicates that Kyn is a product of Trp degradation in pathological conditions, such as DM, and is therefore a disease indicator.

## 5. Conclusions

Evaluation from *Ganoderma lucidum* extract in STZ-hyperglycemic rats indicated that the extract possesses hypoglycemic and hypolipidemia activities. It is important that more Trp was produced in the blood and that it can the improve health status.

## Figures and Tables

**Table 1:** Concentration of beta-glucan, free sugar, proteins and phenols (mg/1 g mushroom) in extract of *Ganoderma lucidum*.

Sample	Beta-Glucan (1,3; 1–6)	Free Sugar Alfa/Beta Glucan	Proteins (BSA)	Phenol (Chlorogenic Acid)
Ganoderm (GWA)	97.2 ^a,^*	25.80 ^a,^*	0.75 ^a,^*	1.35 ^a,^*

* Mean from five samples of fungi. ^a^ Letter in columns indicates that all value in samples were the same. Test Tukey (ANOVA).

**Table 2:** Plasma biochemical analysis from rats submitted to treatments.

Treatments *	Total Cholesterol (mg/dL)	TAG (Triglycerides) (mg/dL)	Urea (mg/dL)	Creatinine (mg/dL)
C	74 ± 10	91 ± 18	32 ± 3	0.55 ± 0.11
DM1	101 ± 3	102 ± 40	57 ± 2	0.83 ± 0.09
CE	60 ± 6	77 ± 23	36 ± 3	0.65 ± 0.01
DM1 + GWA	73 ± 12	98 ± 4	45 ± 2	0.30 ± 0.09
statistic	C and CE *p* < 0.05 DM1 and DM1 + GWA *p* < 0.01	Tendency to decrease	DM1 and DM1 + GWA *p* < 0.01	DM1 and DM1 + GWA *p* < 0.01

* C: Normoglycemic control that received water by gavage; CE: Normoglycemic control group that received hydroethanolic extract (GWA) extract; DM1 + GWA: diabetic group that received extract; and DM1: diabetic group that received water.

**Table 3:** Measurements of islets of Langerhans from rats submitted to treatments.

Treatments	Mean Islet Area (um^2^) *	Mean Number of Nuclei per Islet
C	192.56	69.5 **
DM1	3.40	15 ***
CE	112.84	69.8 **
DM1 + GWA	96.50	88.5 **

* mean of 5 rats. Legend: C = control; DM1 = diabetic; CE = Control + Ganoderma extract; DM1 + GWA = diabetic + Ganoderma extract. ** Statistically nonsignificant; *** statistically significant ANOVA, level of significance = 0.05.

**Table 4:** Composition of amino acids present in plasma of rats submitted to treatments, evaluated by HPLC.

	?	Kyn	Trp
Treatments	Rt = 1.30	Rt = 2.09	Rt = 2.18
C	77.81 *	x	58.8 ug
DM1	77.25	48.81 uM	57.24 ug
CE	121.2	x	101.38 ug
DM1 + GWA	74.02	x	139.71 ug

* Height peak at Rt (Retention time), mean of 3 rats Statistically nonsignificant. ? = Rt (retention time) amino acid unknown perhaps rest of albumin and result presented peak height in mV (millivolt).
